# Proinflammatory cytokine levels, cognitive function, and suicidal symptoms of adolescents and young adults with major depressive disorder

**DOI:** 10.1007/s00406-024-01780-5

**Published:** 2024-03-16

**Authors:** Mu-Hong Chen, Ya-Mei Bai, Ju-Wei Hsu, Kai-Lin Huang, Shih-Jen Tsai

**Affiliations:** 1 Department of Psychiatry, Taipei Veterans General Hospital, No. 201, Sec. 2, Shih-Pai Road, Taipei, 112 Taiwan; 2 Department of Psychiatry, College of Medicine, National Yang Ming Chiao Tung University, Taipei, Taiwan; 3 Institute of Brain Science, National Yang Ming Chiao Tung University, Taipei, Taiwan

**Keywords:** Suicide, Adolescents, Youths, Proinflammatory cytokine, Cognitive function

## Abstract

Whether proinflammatory cytokine dysregulation and cognitive dysfunction are associated with suicidal symptoms in adolescents and young adults with major depressive disorder (MDD) remains uncertain. We assessed the cognitive function and proinflammatory cytokine levels of 43 and 51 patients aged 15–29 years with MDD and severe and mild suicidal symptoms, respectively, as well as those of 85 age- and sex-matched healthy controls. Specifically, we measured serum levels of C-reactive protein, tumor necrosis factor-α (TNF-α), interleukin-2, and interleukin-6 and assessed cognitive function by using working memory and go/no-go tasks. The severity of the patients’ suicidal symptoms was based on Item 10 of the Montgomery–Åsberg Depression Rating Scale; scores of ≤ 2 and ≥ 4 indicated mild and severe symptoms, respectively. The patients with MDD and severe suicidal symptoms had higher levels of C-reactive protein (*p* = .019) and TNF-α (*p* = .002) than did the patients with mild symptoms or the healthy controls. The number of errors committed on the go/no-go by patients with MDD and severe suicidal symptoms (*p* = .001) was significantly higher than those by patients with MDD and mild symptoms or by controls. After adjusting for nonsuicidal depressive symptoms, we observed suicidal symptoms to be positively associated with TNF-α levels (*p* = .050) and errors on the go/no-go task (*p* = .021). Compared with mild suicidal symptoms, severe symptoms are associated with greater serum levels of proinflammatory cytokines and inferior cognitive function in adolescents and young adults with MDD.

## Introduction

The prevalence of suicide mortality in Taiwan peaked in 2006 (19.3/100,000) [1, 2]. Taiwan implemented a suicide prevention program in 2005; in subsequent years (2008–2011), the prevalence of suicide mortality declined to 15.1/100,000 [1, 2]. However, the prevalence of suicide mortality rebounded to approximately 16/100,000 in 2012 and remained at this level up to 2019 [1, 2]. The suicide mortality rate among adolescents and young adults has increased yearly, from 6.1/100,000 in 2008 to 9.1/100,000 in 2019, becoming the second most common cause of mortality in this age group [3, 4]. In 2013, a study of a nationally representative sample of 2,835 college students from Taiwan revealed that approximately 12% and 9% of female and male students, respectively, had attempted suicide at least once in the preceding 12 months [5].

Evidence suggests the crucial role of proinflammatory cytokines in suicidality, including suicidal thoughts, suicide attempts, and suicide mortality [6, 7]. A meta-analysis of 583 patients with suicidality, 315 patients without suicidality, and 845 healthy controls revealed that levels of interleukin (IL)-6 in the blood (serum or plasma) or postmortem brain samples of patients with suicidality were higher than those in the blood of the other two groups [6]. O’Donovan et al. demonstrated that patients with major depressive disorder (MDD) and high suicidal ideation had higher serum levels of IL-6, C-reactive protein (CRP), and tumor necrosis factor-α (TNF-α) than did those with MDD and low suicidal ideation [8]. Miná et al. discovered that the serum concentrations of IL-6, TNF-α, and interferon-γ were increased in both individuals who had attempted suicide and individuals who died by suicide [9]. Isung et al. discovered that plasma IL-6 level was positively associated with impulsivity symptoms as well as violent suicide attempts [10]. However, the aforementioned studies did not adjust for nonsuicidal depressive symptoms when analyzing the associations between proinflammatory cytokines and suicidal symptoms [7–10]. In addition, most patients in these studies were middle-aged adults [6–10]. Thus, whether proinflammatory cytokines are independently associated with suicidal symptoms in adolescents and young adults requires further investigation.

Cognitive dysfunction is a risk factor for suicidality [11–13]. One study investigated 52 and 25 youths with depression who had and had not attempted suicide within the preceding 1 year, respectively; compared with those who had not attempted suicide, those who had attempted suicide exhibited inferior executive function and working memory [12]. A Swedish nationwide cohort study discovered that IQ, an index of cognitive performance, was negatively associated with the risk of suicide attempts in a youth population [11]. The Adolescent Brain Cognitive Development Study determined that the factors that differentiate youths with suicidal thoughts or behaviors from those with mental illness but no suicidal thoughts or behaviors include family conflict, impulse control, depression severity, and a history of mental health treatment [13]. A meta-analysis of 831 patients with major affective disorders and a history of attempting suicide, 824 patient controls with affective disorders only without a history of attempting suicide, and 668 healthy controls discovered that poor performance on trail-making tasks (time to completion) and continuous performance tasks (commission errors) differentiated those with a history of attempting suicide from patient and healthy controls [14]. However, the majority of patients in the aforementioned studies were White. Further studies are necessary to elucidate whether the association between poor cognitive function and suicidality exists among Taiwanese adolescents and young adults.

In this study, we investigated the serum levels of proinflammatory cytokines, namely, CRP, IL-2, IL-6, and TNF-α, and the cognitive function of adolescents and young adults with MDD and mild or severe suicidal symptoms and those of healthy controls. We hypothesized that those with MDD and severe suicidal symptoms would have higher concentrations of proinflammatory cytokines and exhibit greater cognitive deficits than would the healthy controls and patients with MDD and mild symptoms. In addition, we hypothesized that proinflammatory cytokine dysregulation and cognitive dysfunction would be associated with suicidality, independent of depression severity.

## Methods

### Participants

In the current study, we enrolled nighty-four adolescents aged 15–19 years and young adults aged 20–29 years who were diagnosed as having major depressive disorder, current moderate/severe episode, with mild (n = 51) or severe (n = 43) current suicidal symptoms. The Montgomery–Åsberg Depression Rating Scale (MADRS)’s total scores of ≥ 20 were used to characterize the current moderate/severe episodes of major depressive disorder [15]. The mild and severe current suicidal symptoms were defined according to the MADRS item 10 (suicidal thought) scores of ≤ 2 (weary of life; only fleeting suicidal thoughts) and ≥ 4 (feels better off dead; suicidal thoughts common and considered as possible solution but no specific plans/intention), respectively [15, 16]. Exclusion criteria included major medical (i.e., severe autoimmune/immune diseases) or neurological (i.e., stroke, epilepsy) diseases, other severe mental disorders (schizophrenia, bipolar disorder), organic mental disorder, or a history of alcohol or substance use disorders in current study. In addition, patients with a score of 3 on MADRS item 10 were not included in the present study. We also enrolled 85 age- and sex-matched healthy controls without any of the mentioned physical conditions or psychiatric disorders. In all, 43 youths with MDD and severe suicidal symptoms, 51 with MDD and mild suicidal symptoms, and 85 healthy controls were included in current study, with a mean age of about 21 years and a female predominance (Table 1). This study accorded with the Declaration of Helsinki and was approved by the Institutional Review Boards of Taipei Veterans General Hospital. All participants and the parents of adolescent subjects gave their written informed consent.

**Table 1:** Demographic and clinical characteristics between groups

	A. High current suicidal symptom group (n = 43)	B. Low current suicidal symptom group (n = 51)	C. Control group (n = 85)	p value	Post-hoc
Age (years, SD)	21.14 (4.39)	21.90 (4.07)	20.45 (4.93)	0.199	
Female (n, %)	33 (76.7)	38 (74.5)	51 (60.0)	0.081	
BMI (SD)	23.86 (5.58)	21.76 (3.86)	21.58 (3.16)	0.009	A > B ~ C
Education (years, SD)	14.70 (1.91)	14.61 (1.79)	15.48 (2.01)	0.069	
Duration of illness (years, SD)	3.72 (3.56)	3.67 (3.98)		0.205	
Clinical symptoms (SD)					
MADRS total scores	36.07 (4.81)	25.92 (4.00)		< 0.001	
MADRS item 10 scores	4.21 (0.41)	1.25 (0.89)		< 0.001	
History of suicidal attempt (n, %)	26 (60.5)	6 (11.8)		< 0.001	
Treatment with antidepressants (n, %)	39 (90.7)	27 (52.9)		< 0.001	
Treatment with mood stabilizers (n, %)	5 (11.6)	0 (0.0)		0.018	
Treatment with atypical antipsychotics (n, %)	19 (44.2)	15 (29.4)		0.196	

*SD* standard deviation, *MADRS* Montgomery–Åsberg Depression Rating Scale, *BMI* body mass index

### Assessment of inflammatory markers

Inflammatory cytokines, including CRP (QK1707, Human C-Reactive Protein/CRP QuicKit ELISA), IL-2 (QK202, Human IL-2 Quantikine QuicKit ELISA), IL-6 (QK206, Human IL-6 Quantikine QuicKit ELISA), and TNF-α (QK210, Human TNF-alpha Quantikine QuicKit ELISA), in all subjects were assayed using enzyme-linked immunosorbent assay (ELISA) kits (R&D Systems, Minneapolis, MN, USA). Fasting serum samples were collected in serum separator tubes (SSTs) and clotted for 30 min between 9:00AM and 12:00PM. All samples were then stored at − 80 °C until use. All assays were performed according to the manufacturer’s instructions. Final absorbance of the mixture was measured and analyzed at 450 nm using an ELISA plate reader with Bio-Tek Power Wave Xs and Bio-Tek’s KC junior software (Winooski, VT, USA). The standard range depended on the manufacturer’s instructions, and a linear regression, R^2^ value, of ≥ 0.95 represented a reliable standard curve.

### Measurement of neurocognitive functions

In the current study, working memory and go/no-go tasks were examined for working memory and inhibition control. In the working memory task, participants were asked to respond as quickly as possible when they saw a number that appeared again only separated by one other number (i.e., 23–45–23; subjects responded to the second 23 as quickly as possible). In the go/no-go task, participants were asked to respond as quickly as possible after the × symbol appeared. They were not to press the key when the + symbol appeared. After they completed the pretest with all correct responses, the formal test was then administered to record their correct responses, errors, and reaction times (mean). Working memory and go/no-go tasks were commonly used in our previous studies [17, 18].

### Statistical analysis

For between-group comparisons, the F-test was used for continuous variables and Pearson’s test was used for categorical variables. General linear models (GLMs) with adjustment of demographic data (age, sex, BMI) and duration of illness were performed to examine the levels of inflammatory markers (IL-2, IL-6, TNF-α, and CRP) between groups. GLMs with additional adjustment of education were used to examine the cognitive function (working memory and go/no-go tasks) between groups. Furthermore, two correlation analysis models were performed to investigate the associations between suicidal symptoms and inflammatory markers as well as cognitive function among patients with major depressive disorder. In the model 1, age, sex, BMI, duration of illness, and education were adjusted; in the model 2, nonsuicidal depressive symptoms (total MADRS item 1–9 scores) were additionally adjusted. A two-tailed P value of less than 0.05 was considered statistically significant. All data processing and statistical analyses were performed using the SPSS version 17 software (SPSS Inc., Chicago, IL).

## Results

In our study, patients in the severe suicidal symptom group had a higher BMI (p = 0.009) compared with patients in the mild symptom and those in the control groups (Table 1). There were no participants who met the obesity criteria (BMI ≥ 30) in our study. Patients with MDD and severe suicidal symptoms had higher total MADRS (36.07 ± 4.81 vs. 25.92 ± 4.00, p < 0.001) and MADRS item 10 (4.21 ± 0.41 vs. 1.25 ± 0.89, p < 0.001) scores, and exhibited a greater rate of suicidal attempt history (60.5% vs. 11.8%, p < 0.001) than did those with MDD and mild symptoms (Table 1).

**Fig. 1:**
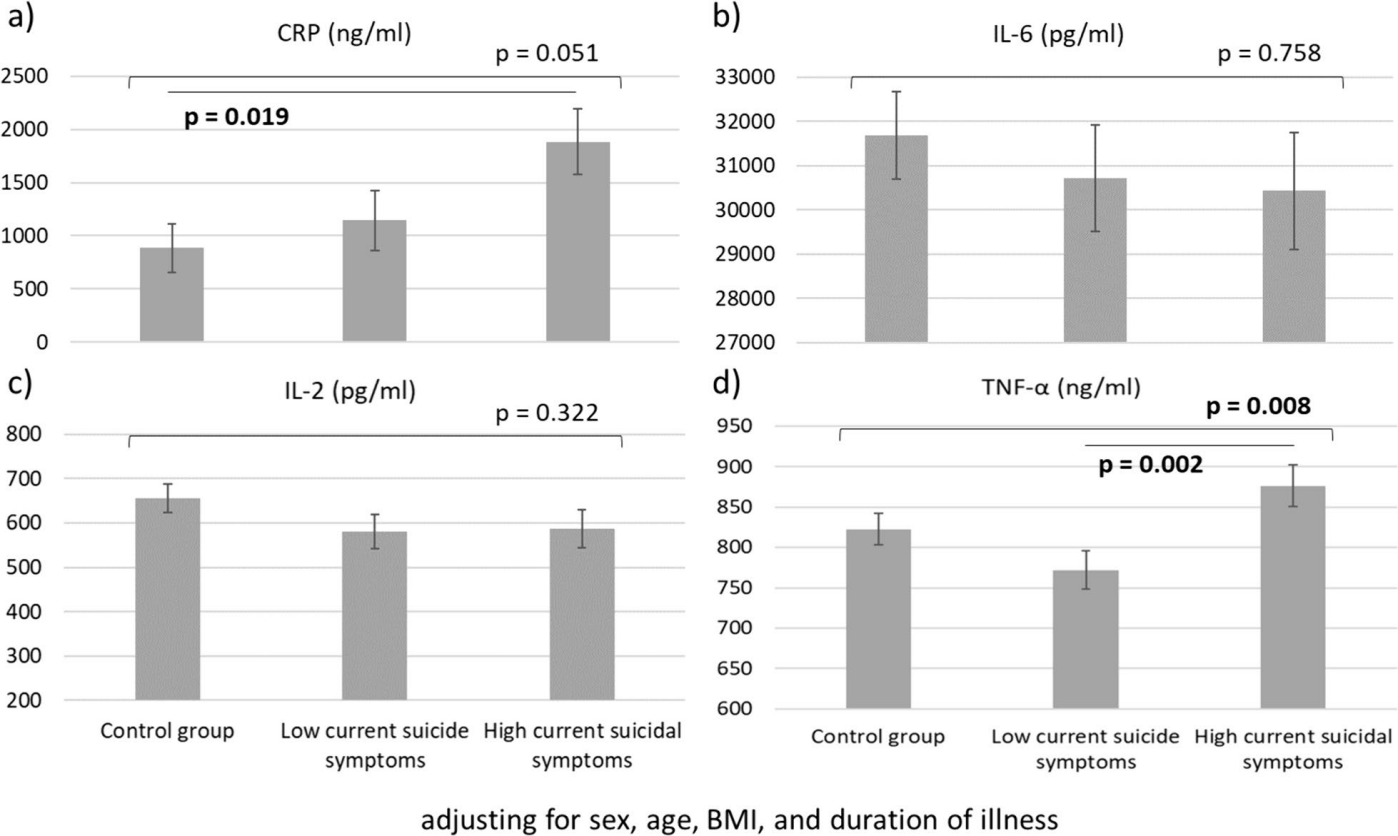
GLMs with adjustment of age, sex, BMI, and duration of illness showed **a** higher CRP levels in the severe suicidal symptom group than in the control group and **d** higher TNF-α levels in the severe suicidal symptom group than in the mild symptom group. There were no differences in **b** IL-6 and **c** IL-2 levels between groups

**Fig. 2:**
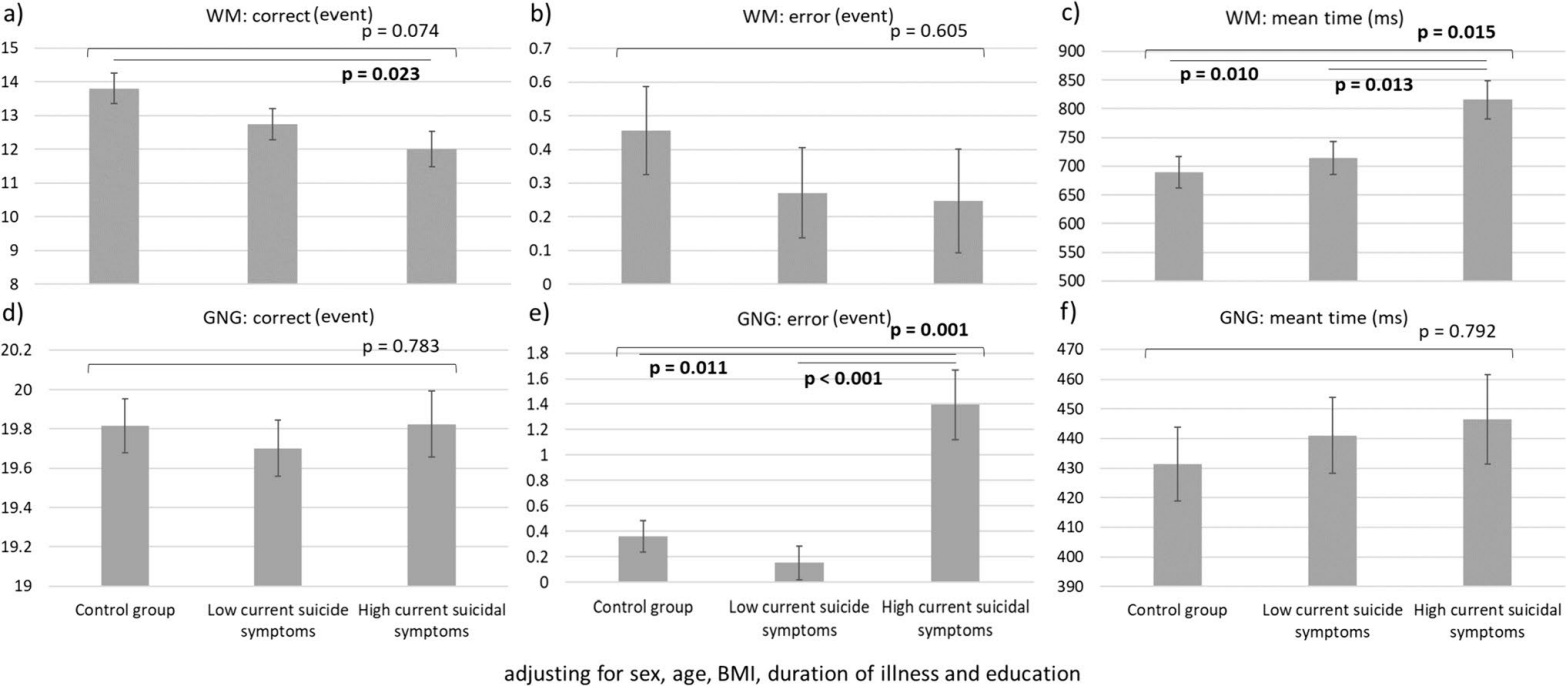
GLMs with adjustment of age, sex, BMI, education, and duration of illness demonstrated that patients with severe suicidal symptoms had **a** lower corrects and **c** a longer mean reaction time in the working memory task compared with the control group. Patients with severe suicidal symptoms exhibited **e** the highest errors in the go/no-go task compared with those with mild symptoms and controls. There were no differences in **b** the errors on the working memory task and **d** the corrects and **f** mean reaction time on the go/no-go tasks between groups

Patients with MDD and severe suicidal symptoms had increased levels of CRP (p = 0.019) and TNF-α (p = 0.002) compared with the controls and those with MDD and mild symptoms, respectively (Fig. 1). Figure 2 showed that the severe suicidal symptom group performed worse on cognitive function tasks, especially mean time on the working memory task (p = 0.015) and errors on the go/no-go tasks (p = 0.001), compared with the mild symptom and control groups. We found no differences (all p > 0.05) in IL-2 and IL-6 levels between groups after adjusting for age, sex, BMI, and duration of illness (Fig. 1). In addition, the errors on the working memory task and the corrects and mean reaction time on the go/no-go tasks did not differ (all p > 0.05) between groups after adjusting for age, sex, BMI, education, and duration of illness (Fig. 1).

Finally, the correlation analysis with adjustment of age, sex, BMI, duration of illness, education, and non-suicidal depressive symptoms found suicidal symptoms (MADRS item 10) were positively associated with TNF-α levels (p = 0.050) and errors on the go/no-go tasks (p = 0.21) (Table 2).

**Table 2:** Correlation analyses of suicidal symptoms with proinflammatory cytokines and cognitive function among patients with major depressive disorder

MADRS item 10 (suicidal symptoms)	CRP	TNF-α	WM: correct	WM: mean time	GNG: error
Model 1: adjusting for sex, age, BMI, duration of illness, and education					
r	0.237	0.425	− 0.173	0.183	0.407
* p*	0.076	**0.001**	0.197	0.172	**0.002**
Model 2: adjusting for sex, age, BMI, duration of illness, education, and non-suicidal depressive symptoms					
r	0.160	0.264	− 0.191	0.133	0.308
* p*	0.240	**0.050**	0.158	0.327	**0.021**

Bold indicates statistical significance (*p* < 0.05)

*MADRS* Montgomery–Åsberg Depression Rating Scale, *CRP* C-reactive protein, *TNF* tumor necrosis factor, *WM* working memory task, *GNG* go/no-go task, *BMI* body mass index

## Discussion

Our findings supported our hypotheses; specifically, adolescents and young adults with MDD and severe suicidal symptoms had higher serum levels of proinflammatory cytokines (i.e., CRP and TNF-α) and exhibited greater cognitive deficits than did healthy controls. Furthermore, we discovered positive associations of suicidal symptoms (MADRS Item 10) with serum TNF-α level and errors on the go/no-go task, independent of nonsuicidal depressive symptoms.

Proinflammatory cytokine dysregulation and cognitive dysfunction are closely related to suicidality [6, 7, 11–13]. A postmortem study of 24 adolescents who committed suicide and 24 matched control individuals found that mRNA levels of IL-6 and TNF-α were significantly elevated in the prefrontal cortices of the adolescents who committed suicide [19]. Juengst et al. reported that higher cerebrospinal fluid and serum levels of TNF-α were associated with behavioral disinhibition and suicidal symptoms [20]. Melhem et al. discovered that youth inpatients admitted for a suicide attempt had CRP and TNF-α mRNA levels that were higher than those of healthy controls [21]. Our findings echo those of the aforementioned studies, suggesting that adolescents and young adults with MDD and severe suicidal symptoms have higher serum levels of CRP and TNF-α than do those with mild suicidal symptoms or healthy controls. In addition, we identified a positive association between suicidal symptoms and TNF-α level after adjusting for nonsuicidal depressive symptoms, supporting our hypothesis that proinflammatory cytokine dysregulation is independently associated with suicidality.

A recent study of 136 patients at high risk for suicide observed that baseline go/no-go task performance, particularly the number of false alarm errors, predicted suicide attempts during the subsequent 90 days [22]. Errors on go/no-go tasks are significantly associated with impulse control, and poor impulse control is a core psychopathology of suicidality [22, 23]. Harfmann et al. discovered that patients with suicidal ideation—with or without a history of suicide attempts—committed more errors on the go/no-go task than did healthy controls [24]. Furthermore, Huang et al. reported that suicidal thoughts, defined as a score ≥ 2 on Item 3 of the Hamilton Rating Scale for Depression, were associated with deficits in working memory and inhibitory control in adults with MDD [25]. A study of 77 adolescent patients with MDD revealed an association between a history of suicide attempts and poor working memory [12]. Zelazny et al. observed that higher working memory scores were associated with a protective effect against suicidal behaviors in youths with mood disorders [26]. We discovered that adolescents and young adults with MDD and severe suicidal symptoms performed significantly worse on working memory and go/no-go tasks than did those with MDD and mild symptoms or did healthy controls. In addition, the number of errors on the go/no-go task was associated with the severity of suicidal symptoms, independent of nonsuicidal depressive symptoms.

The microglia dysfunction may partially explain our study findings of associations between suicidality, inflammation, and cognitive impairment [27–31]. A postmortem brain study of 78 patients with bipolar disorder, 87 with depression, and 85 controls demonstrated that microglial activation was associated with mood disorder diagnosis and suicide [27]. Zheng et al. further emphasized the crucial role of cytomegalovirus in such associations between neuroinflammation and major affective disorder as well as suicide [27]. Holmes et al. assessed the levels of translocator protein (TSPO), which is upregulated in activated microglia, between patients with major depressive disorder and healthy controls using [11C](R)-PK11195 positron emission tomography [31]. They discovered that whereas TSPO was not increased in patients who did not have suicidal thoughts, it was much higher in those with suicidal thoughts, especially in the anterior cingulate cortex and insula [31]. Goncalves de Andrade et al. hypothesized microglia as a hub for suicide neuropathology, and further suggested intervention targeting neuroinflammation may be a possible therapeutic strategy against suicidality [28].

Our study has several limitations. First, we used only working memory and go/no-go tasks to measure cognitive function. Additional studies are thus required to evaluate the associations of different aspects of cognitive function with suicidality in adolescents and young adults with MDD. Second, the patients did not discontinue their use of psychotropic drugs during our cytokine measurement or cognitive assessment. Allowing patients to continue their medication was an ethical choice intended to prevent the exacerbation of depressive and suicidal symptoms. Furthermore, this choice ensured the realism of the data. However, a drug-free study design would be required to validate our findings. Third, there was a small group of patients who experienced chronic suicidal ideation but did not meet the criteria for major affective disorders, including major depressive disorder, in clinical practice. Further studies would be required to investigate such at-risk people.

In conclusion, severe suicidal symptoms are associated with increased serum levels of the proinflammatory cytokines CRP and TNF-α and inferior cognitive function in adolescents and young adults with MDD. Prospective studies are necessary to elucidate the temporal associations of suicidal symptom severity with proinflammatory cytokine elevation and cognitive function, and such research may help clinicians identify predictive markers of suicidality in this age group.

## Data Availability

The datasets generated during and/or analyzed during the current study are not publicly available due to Taiwan’s clinical trial ethical regulation but are available from the corresponding author on reasonable request.
